# Impact of chronic Immune Thrombocytopenic Purpura (ITP) on health-related quality of life: a conceptual model starting with the patient perspective

**DOI:** 10.1186/1477-7525-6-13

**Published:** 2008-02-08

**Authors:** Susan D Mathias, Sue K Gao, Kimberly L Miller, David Cella, Claire Snyder, Ralph Turner, Albert Wu, James B Bussel, James N George, Robert McMillan, Diane Kholos Wysocki, Janet L Nichol

**Affiliations:** 1 Health Outcomes Solutions, P.O. Box 2343; Winter Park, Florida 32790, USA; 2 Amgen, Inc., One Amgen Center Drive; Thousand Oaks, CA 91320-1799, USA; 3 ICON Clinical Research, Lifecycle Sciences Group, 188 Embarcadero, Suite 200; San Francisco, CA 94105, USA; 4 Evanston Northwestern Healthcare and Northwestern University Medical School, 1001 University Place, Suite 100; Evanston IL 60201, USA; 5 John Hopkins University, 624 North Broadway; Baltimore, MD 21205, USA; 6 Phase V Technologies, Inc., 20 Walnut Street; Wellesley Hills, MA 02481, USA; 7 New York Presbyterian Hospital/Weill Cornell Medical Center, 525 East 68th Street; New York, NY 10021, USA; 8 University of Oklahoma Health Sciences Center, P.O. Box 26901; Oklahoma City, OK 73190, USA; 9 The Scripps Research Institute, 10550 N Torrey Pines Road; La Jolla, CA 92037, USA; 10 University of Nebraska at Kearney, Copeland Hall 120B; Kearney, NE 68849, USA

## Abstract

**Background:**

Immune thrombocytopenic purpura (ITP), a condition characterized by autoimmune-mediated platelet destruction and suboptimal platelet production, is associated with symptoms such as bruising, epistaxis, menorrhagia, mucosal bleeding from the gastrointestinal and urinary tracts and, rarely central nervous system bleeding. The aim of this research is to develop a conceptual model to describe the impact of ITP and its treatment on patients' health-related quality of life (HRQoL).

**Methods:**

A literature search and focus groups with adult ITP patients were conducted to identify areas of HRQoL affected by ITP. Published literature was reviewed to identify key HRQoL issues and existing questionnaires used to assess HRQoL. Focus group transcripts were reviewed, and common themes were extracted by grouping conceptual categories that described the impact on HRQoL.

**Results:**

The literature synthesis and themes from the focus group data suggest that decreased platelet counts, disease symptoms, and treatment side effects influence multiple domains of HRQoL for ITP patients. Key areas affected by ITP and its treatments include emotional and functional health, work life, social and leisure activities, and reproductive health.

**Conclusion:**

ITP affects various areas of HRQoL. This conceptual model will help inform the evaluation of therapeutic strategies for ITP.

## Background

Immune thrombocytopenic purpura (ITP) is an autoimmune disorder characterized by accelerated platelet destruction and suboptimal platelet production that leads to reduced peripheral blood platelet counts [1-3]. The etiology of ITP is poorly understood [4]. The estimated annual incidence of adult ITP ranges from 0.6 to 6.6 cases per 100,000 adults [1,4-6]. Women are affected disproportionately, with a female to male ratio of nearly two to one [1]. ITP in adults infrequently remits spontaneously [1] and, although the course of the disease is unpredictable, ITP is rarely fatal, if appropriately managed [4,7].

The physical signs and symptoms of ITP can vary by patient. Some patients suffer from major bleeding that requires immediate attention [8], while other patients with ITP present with few symptoms apart from an increased tendency to bruise or have mucosal bleeding. The degree of bleeding throughout the course of the disease is largely dependent on the patient's platelet count, although other factors certainly contribute. Those with the lowest platelet counts are at the greatest risk for bleeding which can include menorrhagia, gastrointestinal or urinary tracts bleeding and, in rare cases, central nervous system or intracranial bleeding [8]. ITP patients with persistently very low platelet counts (<10 × 10^9^/L) despite treatment, are at risk for both fatal and non-fatal bleeding events [9]. Specifically, Cohen, et al. estimated that five-year mortality rates for ITP patients with persistent low platelet counts (<30 × 10^9^/L) ranged from 2.2% for patients under 40 years of age and 47.8% for patients older than 60 [7].

Treatments currently approved for use in ITP include corticosteroids, intravenous immunoglobulins (IVIG), anti-D immunoglobulins, splenectomy, rituximab, and cyclophosphamide [1,2,10,11]. Standard first line therapy for those with low platelet counts consists of medications such as oral corticosteroids and intravenous immunoglobulins. Patients who do not respond to medical therapies, who relapse after response to therapies, or who require potentially intolerable doses of medical therapies to achieve platelet counts high enough to prevent bleeding usually undergo splenectomy, if the patient is a suitable candidate [11]. Patients who do not respond to or relapse after splenectomy may be treated with a wide spectrum of treatments including corticosteroids, rituximab, danazol, immunosuppressants (e.g. cyclosporine or mycophenolate mofitil) or cytotoxic agents (e.g. cyclophosphamide or azathioprine), each with their own side effects [1,2,4,8]. These treatments have variable effectiveness in treating ITP, and may often be associated with substantial side effects [4]. The one year incidence of diabetes, obesity, and gastrointestinal bleeds are two times higher, and the one year incidences of myocardial infarction and osteoporosis are three times higher in ITP patients receiving treatment with corticosteroids than age- and gender-matched non-ITP patients [12].

Although splenectomy results in long-term disease control in about two-thirds of patients, the long-term outcome in the individual patient is unpredictable [13]. Further, splenectomy patients are at a slightly increased risk for overwhelming sepsis [13]. Currently approved medical treatments for ITP can cause major adverse reactions [14]. For instance, rituximab infusions may cause chills, fever, or severe anaphylactoid reactions [2]; danazol is associated with rash, masculinizing symptoms and liver toxicity [1]; cytotoxic agents may cause cytopenias, gastrointestinal symptoms and, rarely secondary malignancies [8]; and immunosuppressive agents can subject the patient to increased risk of infection [1]. Patients and physicians need to consider both the impact of symptoms of ITP as well as the impact of treatment side effects when making decisions about treatment. It is, therefore, important to incorporate the patient's perspective in decisions regarding the management of their ITP.

Patient-reported outcomes (PROs) provide information from the patient's perspective. PROs have become important tools for understanding the effects of both disease and treatments for various diseases. Health-related quality of life (HRQoL) is the most commonly assessed PRO in clinical research. Both the US Food and Drug Administration (FDA) and the European Agency for the Evaluation of Medicinal Products (EMEA) have emphasized the value of PRO measures in identifying and quantifying the impact of a disease or its treatments on daily life, physical, psychological and social functioning, and well-being [15,16]. Further, these agencies have recently indicated that developing an appropriate and clearly defined conceptual model is a critical step in the development and use of PRO measures [15,16].

In an earlier paper, Mathias, et al. presented the development and validation of an instrument, the ITP-Patient Assessment Questionnaire (ITP-PAQ), to assess HRQoL in ITP patients [17,18]. However, the conceptual model linking the biological and physiological variables of ITP to HRQoL was not included. The aim of this research is to develop a conceptual model to describe the impact of ITP and its treatment on patients' HRQoL.

## Methods

This project uses the model proposed by Wilson and Cleary [19] as a guide for illustrating how biologic and physiologic variables can lead to changes in general health perceptions and overall HRQoL in patients with ITP. Two data sources were used: existing literature and patient contribution. Two literature searches were conducted to identify existing research that gathered information directly from ITP patients regarding their disease or treatment. Also, focus groups were held to solicit patients' input. The focus group format allowed for dynamic responses that built upon the contributions of each patient. Both data sources were referenced in developing a comprehensive conceptual model for HRQoL in adult ITP patients.

### Literature review

The purpose of the literature reviews was to summarize relevant issues for ITP patients, identify existing HRQoL questionnaires used with ITP patients, describe the clinical aspects of chronic ITP, and describe the impact of ITP and its treatments on patients' HRQoL. Search terms in MEDLINE between the years 1997 and 2007 included "idiopathic thrombocytopenic purpura," "immune thrombocytopenic purpura" or "ITP" and "outcomes," "well being," "quality of life," or "questionnaire". The searches were limited to adults (>18 years old). Although, a literature search was conducted at the time of the focus groups in 2001, a supplemental search was conducted now to capture more recent work.

Published abstracts were reviewed to identify those that focused on the effect of ITP or ITP treatments. In addition, new PRO questionnaires that were developed after 2001, one of which was the outcome of the focus group interviews employed here, were reviewed for relevance. Research reports that did not focus solely on ITP, e.g. studies on general hematologic disorders, were excluded from the review. All remaining publications were retrieved and reviewed to identify aspects that affect HRQoL, including symptoms of the disease and side effects of treatments.

### Focus groups

#### Patients

In June 2001, moderated traditional (in-person) focus groups were conducted in New York City, NY (NY), Oklahoma City, OK (OK), and San Diego, CA (CA) to identify the important issue areas for ITP patients. The information from these focus groups was initially used to develop the ITP-Patient Assessment Questionnaire (ITP-PAQ), an ITP-specific HRQoL questionnaire that has since been validated [17,18]. For each focus group, patients were recruited at a local academic-based tertiary care center. The study protocol was prepared and approved by a central Institutional Review Board, and all patients provided written informed consent to participate. Patients were eligible if they were at least 18 years old, had active ITP, and were willing to participate in the focus group. Although there were no specific clinical or medical history requirements for participation in the focus groups, clinicians at each site invited patients who had active disease and required treatment and/or frequent monitoring. Each focus group consisted of 7 or 8 patients, with at least one male patient in each group. A trained moderator used a semi-structured interview format to direct the discussion, encourage interaction among members of the focus group, and ask clarifying questions. The focus groups lasted between 2–3 hours and were audio taped and transcribed. Participants were provided with an honorarium for their time.

#### Data analysis

A project team member divided the transcripts from each focus group into individual units of text. The text units were segments of continuous speech ranging in size from phrases to entire paragraphs that referred to some effect of ITP on the individual's life. No analytic software was used for the qualitative analysis.

Although each focus group was transcribed verbatim, the transcription method was not standardized across focus group location. For instance, each time a focus group participant spoke during the OK and CA focus groups, the transcriber identified the participant by a patient identifier. However, none of the NY participants were similarly identified. Therefore, when evaluating the frequency with which individual patients reported a specific theme, only the OK and CA responses were considered. For the NY focus group responses it was simply assessed whether or not patient(s) in the focus group reported the concept.

Each text unit was given equal weight, except in instances where the same patient reported the same concept during the CA and OK focus groups. In such cases, the text units were grouped and counted only once per patient. After the text units were identified and – when possible – categorized by patient, a second project team member grouped the text units into sub-categories by clustering groups of identical text units, or ones that addressed essentially the same concept. The sub-categories were then grouped into primary conceptual categories. A grid was developed to schematically identify common factors among the themes.

### Conceptual model

Although ideally, the development of a conceptual model would precede the development of a questionnaire, in this instance the activities were not carried out in that sequence. The focus groups and literature review that were conducted in 2001 were used for item generation in the development of the ITP-PAQ in the absence of a conceptual model [17]. We used the focus groups and an updated literature review to develop the structure and content of the conceptual model, in an approach similar to the one described as simultaneous concept development by Finfgeld-Connett [20]. The simultaneous concept development approach assumes that the elements of the conceptual model are likely to be closely linked and the relationships should be considered in developing the model.

The common themes identified in the information from the literature and text units from the focus groups drove the formation of the initial conceptual model. The relationship between the sub-categories and primary conceptual categories were based on the patient attributions. The initial conceptual model was submitted for review at a consensus group meeting where the research team and expert consultants and clinicians reviewed the categories and the proposed relationships to refine the interrelationships among the categories. The categories and relationships were then organized into a final conceptual model representing the impact of ITP on HRQoL.

## Results

### Literature review

**Figure 1:**
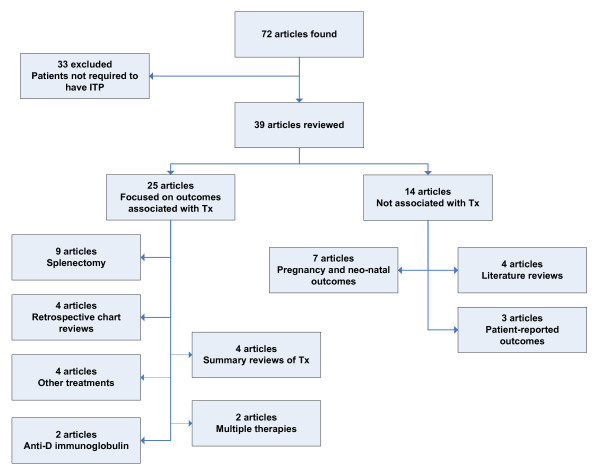
ITP HRQoL literature review flowchart.

The current literature search identified 72 articles of which thirty-three citations were excluded because ITP was not an inclusion criterion for the presented research. Figure 1 describes the categorization of the search results. As illustrated in the figure, most of the articles reviewed focused on the outcomes associated with treatment including comparisons between multiple types of treatments (including corticosteroids) [21,22], open or laparoscopic splenectomy [23-31], intravenous anti-D immunoglobulin [32,33], rituximab [14], intravenous immunoglobulin (IVIg) [34], etanercept [35], combination chemotherapy regimens [36], or summary reviews of treatments [37-40]. The remaining articles pertained to a variety of aspects related to ITP, including pregnancy outcomes [41-47], country-specific retrospective chart reviews [48-51], or literature reviews [13,52-54].

The physical manifestation, the risks, and the clinical aspects of the disease are well characterized in the literature. Some studies mentioned the specific physical signs and symptoms of ITP, such as bleeding and bruising [21,54,55]. The degree, frequency, or severity of bleeding and bruising as a measured outcome of treatment was often reported [25,32,41-43,48,53]. However, the literature search yielded few research reports that collected data directly from the patients or that described the impact of disease on the patients' HRQoL. We describe each study identified in the literature search that incorporated PRO data in characterizing ITP.

In a 2002 study of 28 ITP patients receiving repeated infusions of anti-D, Cooper, et al. asked patients to complete a study-specific six-item questionnaire to assess whether there was a change in HRQoL from the baseline assessment [32]. The six questions included one item on each of the following concepts: well-being compared to baseline, general health compared to the previous year, interference of physical or emotional problems with social activities, and three items related to energy including how often the patient "has a lot of energy", "feels full of pep", and "feels tired". At the 6-month follow-up assessment, patients reported a decrease in well-being compared to their baseline response. At the 12 and 18-month follow-up assessments, patients reported an improvement in general health compared to the previous year, and a reduction in the interference of physical or emotional problems with social activities. There were no significant changes over time in the more specific questions about energy level.

The search also yielded a few articles that focused primarily on the use of patient questionnaires. In 2007, Matzdorff and Arnold surveyed 91 patients with ITP in Germany [55] to determine whether they had been treated according to the 1996 ASH guidelines. Through the survey, the researchers assessed personal and disease history, treatment history, and informational and decision-making history. The most common treatment patients received was corticosteroids (94%), followed by IV Ig (56%). Seventy-five percent of the patients reported experiencing some side effects from corticosteroid treatment, including weight gain (58%), moon face (48%), depression and anxiety (35%), and restlessness and insomnia (22%).

In another study Cohen, et al. modeled quality-adjusted life expectancy in ITP. They made assumptions regarding utility values based on the age- and sex-adjusted Quality of Well Being Index utility values reported for other patient populations, because values were not available for ITP patients. Cohen, et al. predicted that a 30-year old woman remaining thrombocytopenic due to ITP would lose 20.4 years (14.9 quality-adjusted life years) of her potential life expectancy [7].

Zhou, et al. used the Medical Outcomes Study Short Form 36 Health Survey (SF-36) to measure HRQoL in 236 adults with ITP in a cross-sectional study [56]. In addition to the SF-36, a study questionnaire asked patients to rate their fear of bleeding on a 4-point likert scale. Most of the patients (88.1%) reported some fear of bleeding. The authors found that this fear had a significant detrimental impact on SF-36 scores. They also found that patients who had been diagnosed with ITP more than 6 months ago had lower HRQoL than patients diagnosed less than 6 months ago.

Mathias, et al. reported on the development and psychometric validation of the 44 item ITP-PAQ to measure HRQoL [17]. The ITP-PAQ was developed based on findings from the published literature, existing questionnaires, expert clinical opinion from leading hematologists, and input from focus groups of ITP patients. The final questionnaire contains 10 scales, including Symptoms, Bother-Physical Health, Fatigue/Sleep, Activity, Fear, Psychological Health, Work, Social Activity, Women's Reproductive Health, and Overall QOL.

Recently, McMillan, et al. reported on the comparison of SF-36 scores of ITP patients with SF-36 scores of the general US population and the scores of patients with hypertension, arthritis, diabetes mellitus, congestive heart failure, missing or paralyzed limb, or cancer [57]. These authors found significantly lower scores in patients with ITP compared to the general US population for nearly all the SF-36 domains (except mental health). They also found that the physical impact of chronic ITP on HRQoL was similar to that of diabetes and greater than that of hypertension, arthritis, or cancer. They acknowledge that distinguishing whether ITP or the treatment of ITP contributes to the reduction of HRQoL can be challenging, especially with a generic instrument like the SF-36.

### Focus groups

Twenty-three patients (OK:8, CA:7, NY:8) participated in the focus groups, 16 (70%) were women (OK:5, CA:6, NY:5). No additional demographic data were collected. Focus group data used to shape the conceptual model are summarized in Tables 1 and 2. Table 1 contains the comprehensive list of themes reported by the patients. Each theme is grouped into a primary conceptual category (a-g). Table 2 indicates the emphasis given to each primary conceptual category during the focus group discussions. Not surprisingly, based on the patients' attributions (a) symptoms, e.g., bleeding and bruising, that result from low platelet counts; and, (b) side effects of treatment, e.g., weight gain and mood swings, were mentioned as significant impairments to overall HRQoL. These two categories were considered the main determinants of HRQoL changes for patients with ITP.

**Table 1:** Comprehensive list of themes

a. Signs and Symptoms	c. Emotional Health
Fatigue	Relationships
▪ Inability to get out of bed	▪ Spouse
▪ Limits daily activities	▪ Family
Bleeding	▪ Friends
▪ Blood blisters	▪ Children
▪ Bleeding from gums	Fear, Stress, & Anxiety
▪ Nose bleeds	▪ Fear of accidents
▪ Embarrassment	▪ Fear of intracranial bleeding
Bruising	▪ Fear of low platelet counts
▪ Bruises that never go away	▪ Fear of dying
▪ Bruises all over legs	▪ Financial stress
▪ Petechia	▪ Stress contributes to low platelet levels
Other	▪ Anxiety about low platelet levels
▪ Migraines	▪ Anxiety medical profession's lack of knowledge
▪ Visual impairment	Depression, Isolation, oss of Control
▪ Joint aches	▪ Seek therapy or counseling
	▪ Suicidal
b. Treatment Effects	▪ Go off by myself
Steroids	▪ Feels alone
▪ Mood swings	▪ Family/friends don't understand ITP
▪ Weight gain	▪ Resentment towards health professionals
▪ Anger	▪ Feels pressure to be strong
▪ Anxiety	Mood & Self-Conscious
▪ Trouble sleeping/insomnia	▪ Mood swings
▪ Round face	▪ Choice of clothing limited due to bruising
▪ Lump in back	▪ Bothered by needle marks
Other treatments	▪ Avoid mirrors
▪ Headaches	▪ Embarrassed by bleeding
▪ Hair loss	▪ Self-conscious about bruising
▪ More susceptible to colds and fungal infections	
	
d. Functional Health	f. Social and Leisure Activities
Daily Activities	Sports/exercise/physical activities
▪ Housework, including cooking	▪ Unable to go to the gym
▪ Fatigue limits daily activities	▪ Unable to do sports, boxing, martial arts, skydiving, climbing, dancing
▪ Extreme care in doing simple tasks	Leisure activities
Changes in Lifestyle	▪ Unable or too tired to go out with friends
▪ Inability to plan for the future	Travel is limited or more difficult
▪ Reduction in risk-taking activities	Feelings of isolation due to physical and emotional effects of ITP
▪ Hide the severity of disease from family	Social stigma
Sleep	▪ People suspect spousal or parental abuse due to bruising
▪ Restlessness	
▪ Not comfortable	
▪ Tired, but unable to sleep	
	
e. Work Life	g. Reproductive Health
Absences	Women's Reproductive Issues
▪ Permanent disability	▪ Hysterectomy because of bleeding
▪ Frequent absences due to illness, and due to medical visits	▪ Heavy menstrual bleeding
▪ Unemployed	▪ Inability to have children
Change in Attitudes	Sex
▪ Work to support family	▪ Bruising
▪ Work in a low risk environment	▪ Bleeding
▪ Work is a lower priority	▪ Reduced libido
▪ Work is not as satisfying	
Productivity	
▪ Fatigue hinders work	
▪ Working part-time due to absences	
Career advancement	
▪ Lost promotions	
▪ Unable to pursue desired career	
▪ Change career	

**Table 2:** Frequency of Reports of Primary Conceptual Categories and Sub-Categories from ITP Focus Groups

	**OK**	**NY**	**CA**		N = 15 (%)*
**a. Signs and Symptoms**				**14 (93%)**	
Fatigue	+	-	+		14 (93)
Bleeding	+	+	+		8 (53)
Bruising	+	+	+		8 (53)
Other	+	+	+		8 (53)
**b. Treatment Effects**				**13 (87)**	
Steroids	+	+	+		13 (87)
Other treatments	+	+	+		8 (53)
**c. Emotional Health**				**14 (93)**	
Fear, stress & anxiety	+	+	+		11 (73)
Relationships	+	+	+		7 (47)
Depression, isolation, & loss of control	+	+	+		7 (47)
Mood & self-consciousness	+	+	+		7 (47)
**d. Functional Health**				**13 (87)**	
Daily activities	+	+	+		11 (73)
Sleep	+	+	+		9 (60)
Changes in lifestyle	+	+	+		7 (47)
**e. Work**				**13 (87)**	
Absences	+	+	+		10 (67)
Changes in attitudes	-	+	+		5 (33)
Career advancement	+	+	+		3 (33)
Productivity	+	+	+		4 (27)
**f. Social and Leisure**				**10 (67)**	
Sports/exercise/physical activity	+	+	+		5 (33)
Leisure activities	+	+	+		5 (33)
Social Stigma	+	-	+		4 (27)
Travel	+	+	+		3 (20)
**g. Reproductive Health**				**10 (67)**	
Sex	+	+	+		9 (60)
Women's reproductive issues	+	+	+		8 (53)

+ = theme present in focus group

- = theme not present in focus group

* based on data from OK and CA focus groups only

Also, Table 2 provides details about the sub-categories included in each of the primary conceptual categories. The table indicates whether the sub-category was mentioned during each of the focus groups and provides the number (and percentage) of patients that mentioned the sub-categories in the CA and OK focus groups. Although most sub-categories were mentioned during all focus groups, a few differences emerged which are highlighted below.

### Conceptual model

**Figure 2:**
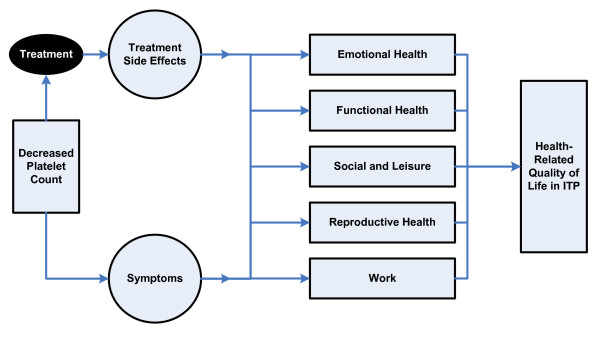
**Conceptual model for HRQoL in patients with ITP**.

Based on the findings from the literature review and focus groups, we developed a conceptual model as presented in Figure 2. Following the Wilson & Cleary model [19], the biologic variable (platelet count) and the main determinants (signs and symptoms of ITP and the treatment effects) were placed on the left side of the causal model. This was followed by the domains of emotional and functional health, work life, social and leisure activities, and reproductive health. This model proposes that symptoms of ITP and side effects of its treatment lead to a worsening in HRQoL by adversely affecting certain domains. Below we discuss each component of the model.

#### Biological variables

Platelet counts, with or without the presence of any signs or symptoms, are often the only determinant of treatment decisions [54,58] and are also used to establish whether the patient has responded to treatment and whether the response is considered complete, partial, or minimal [54]. Platelet counts are inversely correlated with bleeding outcomes, and are of utmost concern to patients. Patients that had undergone splenectomy emphasized that generally their platelet counts rose immediately following the procedure, but then the platelet counts leveled off or dropped in the months and years post-surgery. The potential for and actual drops in platelet counts may cause emotional distress. Some patients report that it is "like a rollercoaster" or that their platelet levels "go way up and then way down". Table 3 contains further examples of verbatim patient quotes on platelet counts and other discussion areas from the focus group participants.

**Table 3:** Direct quotes from ITP patient focus groups

Biological Variables	▪"like a rollercoaster" ▪" [platelet levels] go way up and then way down" ▪ "[The fact that] you're sick now but you'll be okay in 2 days or 2 weeks... was a particularly difficult area for me to handle." ▪ "[After splenectomy] my platelet count went up to 500,000 right after splenectomy, a month later it was back down to the teens."
***Main Determinants***	
Signs & Symptoms	▪ "Fatigue is [the] number one [issue] for me." ▪ "I guess the worst was probably ... when I had a lot of bleeding in my mouth. I hate when I taste blood." ▪ "I'm bruised all the time and I'm pretty used to it now. It's very annoying, but it's just a way of life now." ▪ "I've had about three really heavy duty nosebleeds lasting several days and you just have to immobilize yourself and hope that the clot will finally hold." ▪ "I get bruises now and then. I do have petechia most of the time on my legs"
Treatment Effects	▪ "I won't take it [steroids] and my count is 3 and I won't take it because my quality of life means too much and I already don't feel good." ▪ "I'm very susceptible to illness, colds, and lung problems. I've become fanatical with germs. I don't touch things." ▪ "It's like rocket fuel. All of you know who've done steroids, it's terrible stuff. I think steroids are the worst. steroids were worse than the chemo drugs were." ▪ "If I would have heard 50/50 [chance of success/failure] I wouldn't have done it. That 70/30 sounded really good to me."
***HRQoL Domains***	
Emotional Health	▪ "The thing about families is they look at you and expect to see you being strong. I feel like they expect me to be able to deal with this." ▪ "The worst part about it is the emotional strain that was put on everyone around me." ▪ "There [are] times that I won't go to the doctor because I know that I can't afford to go." ▪ "I have that fear [of accidents]. I always make the sign of the cross because I know if I get banged up I'm gonna bleed." ▪ "When you're in a flare you just feel off in a corner by yourself and you're isolated from everyone."
Functional Health	▪ "I get so tired I can't even move." ▪ "I never sleep through the night. It's just restlessness and you're not comfortable." ▪ "You're limited. You don't do what you used to do to the same degree and you'd still like to do that but you can't do it." ▪ "When cooking...you have to be very careful. You have to really take your time when you're cutting."
Work Life	▪ "I loved working but the fact that I have to sleep when I'm not working makes me want to work less so that I can do something besides sleep." ▪ "Now I'm a technical writer, which has no [bleeding] risk at all. I just sit at the computer and type. I really don't like it, but I don't have too many options." ▪ "I had to take off a lot to go to the hospital to get the treatment." ▪ "I wanted to be a nurse but I couldn't because I had ITP." ▪ "Working part-time."
Social & Leisure Activities	▪ "My friends think I'm crazy because I won't go the movies with them on a Friday night. I'm just so exhausted when I get home. I don't have the energy to get dressed and go to a movie." ▪ "My bruising bothers me especially in the summertime when I can't [go swimming] because I'm always bruising and people look at you funny." ▪ "I've had co-workers and friends ask me if my husband was beating me." ▪ "When you're bruising, especially during the summer, and you have to go outside and you're so self conscious because you know what people are thinking because they automatically just assume, oh boy she's been beat." ▪ "Well, I find myself more reluctant about meeting new people because I have so many bruises so often." ▪ "I don't go jumping off planes. I don't even want to swim in the water. I just don't want to get hurt. I'm very fragile now." ▪ "I said, Don't look at me to go anywhere. I'm not leaving New York." ▪ "I always take out traveler's insurance now for cancellations." ▪ "We used to do extensive traveling and we've limited that down to a week or less at a time any more and we don't do as many of them as we used to." ▪ "With my grandchildren, they want to jump on me. They want to do a lot of things. I want to do a lot of things with them. My daughter tells them 'Don't jump on Grandma. Don't pull her hair. Be careful.' That really bothers me."
Reproductive Health	▪ "When I first got ITP I thought that I couldn't have children. And then my gynecologist told me that I could have children while taking prednisone. All those years my hematologist made me think I couldn't have children." ▪ "You have to be cautious...If I had sexual relations [I was afraid] something was gonna happen to me." ▪ "I don't feel sexy. I don't know if it's the weight gain or if it's the stigma of being sick." ▪ "When my platelets are low I say "Stay away." [regarding sex]" ▪ "Much heavier [periods] and lasted anywhere from 6 to 9 weeks" ▪ "Now on the prednisone my desire has went [sic] down considerably. I swear I used to be sexually active 4 or 5 nights a week, now it's once or twice a month."

The focus group comments regarding the unease associated with platelet counts support Zhou, et al.'s assessment that the fear of bleeding due to low platelet counts may itself interfere with patients' HRQoL [56].

#### Main determinants

##### Signs and Symptoms

Table 2a indicates the most commonly reported signs and symptoms that patients attributed to ITP. One patient in the CA focus group characterized fatigue as follows, "Fatigue is [the] number one [issue] for me." Moreover, as shown in Table 2a, fatigue was reported by over 90% of the patients in the CA and OK focus groups. Interestingly, fatigue was not mentioned in the NY focus group.

Other very commonly reported symptoms were bleeding and bruising. In describing her bleeding, one patient said "I guess the worst was probably ... when I had a lot of bleeding in my mouth. I hate when I taste blood." Another patient described her bruising by stating, "I'm bruised all the time and I'm pretty used to it now. It's very annoying, but it's just a way of life now."

Although they did not provide details or rationale for the selection of the items, Cooper, et al.'s choice of questions suggests that fatigue was a primary concern for ITP patients [32]. Other reports in the literature suggest that anemia, a likely cause of fatigue, is common in ITP patients, especially in the presence of bleeding [48]. In particular, Cines and Bussel provide anecdotal evidence of a young female patient experiencing extreme fatigue at platelet counts <50,000 × 10^9^/L [59].

##### Treatment Effects

Table 2b indicates that the most commonly reported adverse effects of ITP treatment were attributed to the use of steroids. The focus group patients reported numerous effects of corticosteroid treatment, including anger, insomnia, mood swings, and weight gain (see Table 1 for more detail). Most patients in the CA and OK focus groups reported that the adverse effects of steroids negatively impacted their lives. One patient summarized her feelings about steroids by stating, "I won't take it and my count is 3 and I won't take it because my quality of life means too much and I already don't feel good." Some patients also expressed increased concern over susceptibility to infection as a result of splenectomy. For instance, a female patient in the CA focus group stated "I'm very susceptible to illness, colds, and lung problems. I've become fanatical with germs. I don't touch things." Patients also articulated some concern about the decision to proceed with a splenectomy when remission following splenectomy is far from certain. Similar observations were reported in the findings of Matzdorff and Arnold [55].

#### HRQoL domains

##### Emotional Health

Patients frequently mentioned "anxiety", "depression", and "fear" to describe their feelings about their symptoms. Many patients reported that ITP and its symptoms had an effect on their personal relationships. Table 2c shows that emotional health concerns were an issue discussed in all focus groups, with a similar number of patients expressing concern over relationships, mental health, and self-consciousness. The emotional concerns appeared to affect women differently than men. Men were particularly concerned with their ability to provide for their families and their need to appear stoic. In contrast, women discussed the protective nature of family and friends and the effect their illness had on others. This is illustrated by a male participant in the OK focus group commenting, "The thing about families is they look at you and expect to see you being strong. I feel like they expect me to be able to deal with this." A female participant in the same focus group stated, "The worst part about it is the emotional strain that was put on everyone around me." The financial strain triggered by the medical expenses also creates a stressful environment for many patients. One patient stated, "There [are] times that I won't go to the doctor because I know that I can't afford to go."

##### Functional health

Patients reported a strong relationship between the symptoms of ITP and functional health limitations particularly with respect to the need to limit daily activities (see Table 2d). For instance, patients reported that their activities were limited because of fatigue. "I get so tired I can't even move." or "When I've been on my legs too long, they start cramping" were just two of the comments made by focus group participants. Difficulty sleeping was also reported by many focus group patients, with one patient stating, "I never sleep through the night. It's just restlessness and you're not comfortable."

##### Work life

As indicated in Table 2e, the majority of the patients in the focus groups indicated that ITP had interfered with their ability to work, and some individuals mentioned that ITP had hindered their ability to advance in their career. Many patients reported that they took time off or quit working entirely due to the symptoms or treatment of ITP. A female patient stated, "I loved working but the fact that I have to sleep when I'm not working makes me want to work less so that I can do something besides sleep." Patients in CA and NY reported that they had experienced a change in their desire or reason for going to work. For instance, one patient stated, "Now I'm a technical writer, which has no [bleeding] risk at all. I just sit at the computer and type. I really don't like it, but I don't have too many options." However, no patients in the OK focus groups mentioned a change in attitude toward work or work environments.

##### Social and leisure activities

Table 2f shows that patients with ITP report suffering from feelings of social embarrassment due to visible signs of the disease (bruising) and that their involvement in sports or other physical activities is limited. Testimony such as "My bruising bothers me especially in the summertime when I can't [go swimming] because I'm always bruising and people look at you funny" and "I've had co-workers and friends ask me if my husband was beating me" were recorded. Patients also reported that symptoms of ITP prevent them from participating in leisure activities such as exercise, gardening, and travel. A female patient commented, "My friends think I'm crazy because I won't go [to] the movies with them on a Friday night. I'm just so exhausted when I get home. I don't have the energy to get dressed and go to a movie."

##### Reproductive health

Both male and female participants in the focus groups reported decreased libido due to the symptoms of ITP and the side effects of treatment. A male participant stated, "Now on the prednisone my desire has gone down considerably. I swear I used to be sexually active 4 or 5 nights a week, now it's once or twice a month." Female participants also reported bruising and bleeding as a result of sexual intercourse.

The numerous publications dealing with pregnancy and obstetrics indicate that ITP greatly affects women, particularly with respect to child bearing. Information from the focus groups highlighted the exacerbation of menstrual bleeding (both severity and duration) in women with ITP. In addition, some women also mentioned anxiety over the potential inability to bear children. However, the literature suggests that the outcome of pregnancy in women with ITP is generally good, if close monitoring and treatment is provided to expectant mothers and infants [41]. One woman gave the following anecdote that was characteristic of other reports: "When I first got ITP I thought that I couldn't have children. And then my gynecologist told me that I could have children while taking prednisone. All those years my hematologist made me think I couldn't have children."

## Discussion

The clinical manifestations of ITP and its management affect patients' everyday activities and well-being. Our research aimed to propose a conceptual model that describes the impact of ITP and its treatments on patients' HRQoL by using information gathered from the published literature and from the patient perspective reported in focus groups. Qualitative methods were used to group the patient reports into one biological variable, two main determinants, and five conceptual domains of HRQoL relevant to patients with ITP.

A conceptual model providing a proposed causal linkage with HRQoL can be useful for several reasons. To begin, it helps to further explore the disease area and proposes a pathway for how benefits and risks of new treatments may impact the lives of ITP patients. Also this pathway will assist researchers in selecting (or developing) appropriate outcome measures to evaluate a treatment's efficacy.

Although the etiology of ITP is poorly understood, patients with low platelet counts are at greater risk of complications. Also, platelet counts, with or without the presence of symptoms, can dictate whether to treat ITP. It is to be expected, then, that in the focus groups, patients reported keen awareness and close monitoring of the ups and downs of their platelet counts. The emphasis placed on the fluctuations in this clinical marker appears to cause nearly as much anxiety for the patient as the actual disease symptoms, particularly for patients that have undergone splenectomy with the hope of full permanent remission. Further, the focus group patients reported some distress about the confusion surrounding the disease, specifically with relation to childbearing. Future efforts to educate those affected by the disease could reduce the discrepancy between the perceptions and the medical facts.

Symptoms of the disease were found to be important factors characterizing patient well-being. According to patients, the bruising and bleeding resulting from ITP significantly worsen HRQoL. In addition, since fatigue substantially hindered patients' ability to perform their daily activities, the management of fatigue could potentially improve overall HRQoL. However, despite our finding that over 90% of the patients in the CA and OK focus groups mentioned fatigue, currently fatigue is not sufficiently often considered by clinicians who manage persons with ITP. In fact, fatigue is seldom recorded as an adverse event in clinical trials of ITP patients.

In addition to potentially severe clinical outcomes, the effects of the various ITP treatments impact multiple facets of the patients' lives. However, the adverse effects of corticosteroids, such as weight gain and mood swings, were most emphasized during the focus group discussions. Patients reported side effects of other treatments, such as hair loss and susceptibility to infection, less frequently. Since treatment with corticosteroids is usually first-line therapy for patients with ITP, it is likely that all patients in the focus groups had received treatment with corticosteroids at some time.

Patients reported some concern over increased susceptibility to infection as a result of splenectomy. These reported fears may seem unfounded when reviewing some reports of high success rates (*i.e*., increased platelet counts) one year following splenectomy [21,26]. However, some evidence suggests that longer-term outcomes may not be as favorable bringing to question the risk-benefit ratio of splenectomy in the face of all the anxiety. Kojouri, et al. found that 66% of the patients had a complete response to splenectomy, defined as achievement and maintenance of a normal platelet count, (> 150 × 10^9^/L or as defined in the original report and > 100 × 10^9^/L for all measurements 30 days or longer after splenectomy, and with no additional treatment for ITP, except for the tapering of perioperative glucocorticoids or other treatments) with a median follow-up of 29 months. They also found surgical complication rates of 12.9% and 9.6% for laparotomy and laparoscopic splenectomy, respectively. However, they acknowledge that the follow-up duration may not have been long enough to provide a valid assessment of long-term risks associated with splenectomy, especially since relapse rates increase with duration of follow up [13]. Portielje, et al. found that, of those patients who had experienced complete response (platelet count >100 × 10^9^/L) within 2 years of diagnosis, 45% had experienced at least one ITP-related hospital admission in the 10-year follow-up period [49]. McMillan and Durette studied 105 chronic ITP adults who were refractory to splenectomy. During the median follow-up period of 110 months, 6 patients (5.6%) died of treatment-related complications (including sepsis associated with long periods of immunosuppression, postoperative pancreatitis, and transfusion-related hepatitis C) [23].

It is worth noting that just as the model provides a pathway for describing how negative outcomes or adverse events of treatment affect the patients' HRQoL; the model also holds in the case of positive effects of treatment. Although it was not emphasized explicitly in the focus groups, any effects of a particular treatment that patients perceive as positive (e.g., a stable platelet count, increase in energy and vitality, decrease in anxiety) would likely improve the domains of HRQoL.

ITP researchers have acknowledged the value that PRO measures would bring to understanding the signs and symptoms of ITP and its treatments effects on HRQoL. After conducting a literature review aimed at assessing variability and terminology used in the diagnosis and treatment of ITP, Ruggeri, et al. suggested that further studies consider the effect of treatment options on quality of life evaluations as well as treatment effects on platelet counts [54]. Bussel, et al. also recognized that improved treatment outcomes with new treatments can have an effect on patient HRQoL [34]. Our literature searches yielded only four instances in which a questionnaire was used to assess quality of life [17,32,56,57]. The PRO endpoints pursued in the studies were consistent with the concerns voiced by the patients in the focus groups, e.g. fear of bleeding and extreme fatigue.

A multi-dimensional measure of HRQoL in ITP can be particularly useful when comparing HRQoL outcomes of treatments that may have differential impacts on HRQoL or when some aspects of HRQoL may be improved at the expense of others. Our proposed conceptual model delineates concepts that may be considered in a PRO questionnaire to assess HRQoL changes in persons with ITP. Most of these concepts are included in the ITP-PAQ; any differences between the model and the ITP-PAQ reflect that the questionnaire was developed for use in clinical trials and could not contain all issues that may be relevant to patients. The model will also assist in the selection of items, scales, and/or questionnaires that may be appropriate to utilize when assessing HRQoL in ITP patients.

This research has some limitations. For one, the conceptual model focuses specifically on the relationship between symptoms, side-effects, and HRQoL and did not address other concepts such as treatment satisfaction, treatment decision-making, and medication compliance. Research looking into the roles of these other outcomes on patient HRQoL in ITP may be important.

The decision to use focus groups to gather patient contributions also created limitations. For instance, only English-speaking patients in the US were sampled; therefore the conceptual model may need further testing to be generalizable in other populations. The time commitment required for focus group participation and the use of an honorarium as an incentive for participating may have introduced a self-selection bias into the sample population. Also, the entry criteria for participation in the focus groups were very broad. No strata were used to guarantee a distribution in severity of disease, success of treatment, duration of disease, or age. All patients were recruited by physicians from tertiary care centers that tend to treat more severe or refractory patients. Therefore, this sample may be biased by over representing patients that are, in general, more ill or more aware of their illness. It is worth noting that although group interaction can stimulate participant ideas that might not have been available on an individual basis, it may also lead to overemphasis of the opinions of the groups' participants. For this reason, it is critical to the success of a focus group to have a highly experienced moderator guide the discussion.

Finally, because we could not match demographic or clinical history to individual focus group participants, it was not possible to distinguish whether the concepts affected some patients more than others. In particular, gender and age may play an important role in determining the HRQoL of adult patients living with chronic ITP. Further studies on the varying severity and progress of ITP may lead to slightly different conclusions regarding the pathway from platelet counts through signs and symptoms and HRQoL domains to overall HRQoL. However, the intent of this research was to develop a broad conceptual model.

Future research will build on the findings of this study. The sample size needed for qualitative research is determined by the concept of theoretical saturation. In order to achieve saturation, data must be collected until no new information is obtained. For this model our empirical data source, the existing transcripts of contributions from 23 patients, was not designed to reach saturation of themes or concepts. Researchers may wish to collect additional patient data either in focus groups or individual interviews to reach saturation on all areas of interest.

While the literature searches yielded several questionnaires that have been used to assess HRQoL in persons with ITP, only the ITP-PAQ, developed based on the feedback from these very focus groups of ITP patients, was designed and validated in patients with ITP. The ITP-PAQ covers many of the themes identified in our conceptual model for ITP, however other concepts important to patients, e.g. ability to perform daily activities, feelings of social isolation, inability to travel, financial stress, and reduction in libido, are not included in the ITP-PAQ. Patient-reported data captured with questionnaires such as the ITP-PAQ may be used to refine the conceptual model presented here. We expect further targeted research, in the style of Moore, et al. [60], to be used to test the relationships proposed in the ITP conceptual model, e.g., observing the changes in HRQoL of a subset of patients with a specific treatment or patients with a specific symptom.

## Conclusion

A conceptual model of health-related quality of life for patients with ITP consisting of two main determinants was developed: (1) signs and symptoms; and, (2) treatment effects; and five HRQoL domains: (1) emotional health, (2) functional health, (3) work life, (4) social and leisure activities, and (5) reproductive health. This conceptual model should help to inform the evaluation of therapeutic strategies for ITP.

## Competing interests

The analysis of focus group transcripts, interpretation of results, conceptual model design, and the writing of the manuscript represent the joint collaboration of all authors of this study, which was funded solely by Amgen, Inc, Thousand Oaks, California, USA. No other additional funding for this study was provided. The decision to submit this manuscript for publication was subject to the approval of Amgen, Inc. and all authors.

SDM is the President of Health Outcomes Solution. SKG and JLN are employees of Amgen, Inc. KLM is an employee of ICON Clinical Research Lifecycle Sciences Group. DC is employed by Northwestern University Medical School. CS is employed by John Hopkins University, School of Medicine. CS' contribution to this publication was as a paid consultant to Amgen. AW is employed by John Hopkins University, Bloomberg School of Public Health. RT is an employee of Phase V Technologies. JBB is an employee of Weill Cornell Medical Center. JNG is an employee by the University of Oklahoma Health Sciences Center. RM is a Professor Emeritus of the Scripps Research Institute. DKW is an employee of the University of Nebraska at Kearney.

## Authors' contributions

SDM supervised the interpretation of the results from the focus groups and conceptual model development activities and assisted in the drafting of the manuscript. KLM reviewed the focus group transcripts and participated in the drafting the manuscript. JBB, JNG, RM, and JLN provided clinical expertise in the design and execution of the focus groups and in the drafting of the clinical aspects of the manuscript. DC, CS, RT, and AW provided outcomes expertise in the development of the conceptual model and participated in the drafting of the manuscript. DKW provided her expertise in the design, execution, and moderating of the focus groups, and in the review of the manuscript. SKG assisted in interpreting the results and drafting the manuscript. All authors contributed to the development of the manuscript. All authors read and approved the final manuscript.
